# Comprehensive mini-review: therapeutic potential of cannabigerol – focus on the cardiovascular system

**DOI:** 10.3389/fphar.2025.1561385

**Published:** 2025-03-26

**Authors:** Anna Krzyżewska, Monika Kloza, Hanna Kozłowska

**Affiliations:** Department of Experimental Physiology and Pathophysiology, Medical University of Białystok, Białystok, Poland

**Keywords:** hypertension, phytocannabinoids, animal models, oxidative stress, inflammation, alpha-2-adrenergic receptors

## Abstract

**Backgrounds:**

Cannabigerol (CBG) is a non-psychoactive phytocannabinoid with a broad spectrum of biological effects. However, there is still too little research on its safety especially its effects on the cardiovascular system. Due to its agonist effects on alpha-2-adrenergic receptors (α_2_AR), it is speculated that it may have applications in the pharmacotherapy of metabolic syndrome, particularly hypertension. Thus, the aim of our review was to analyse the therapeutic potential of CBG in cardiovascular diseases.

**Methods:**

The review was based on searches of the PubMed and Web of Science databases. Keywords were used to identify literature containing therapeutic and mechanistic information on CBG and its potential effects on the cardiovascular system.

**Results:**

A review of the literature shows that CBG exhibits hypotensive effects in mice probably through α_2_AR agonism. Other numerous *in vitro* and *in vivo* studies show that CBG has anti-inflammatory, antioxidant effects and also regulates cell apoptosis. Cannabigerol improved tissue sensitivity to insulin, and also showed efficacy in inhibiting platelet aggregation. However, there are reports of adverse effects of high doses of CBG on liver architecture and function, which calls into question its usefulness and safety profile.

**Conclusion:**

Above mentioned beneficial properties of CBG suggest that it may be useful in treating hypertension and metabolic syndrome. However, there is still a lack of studies on the chronic administration of CBG and its effects on cardiovascular parameters in hypertension condition, which may be necessary to determine its safety and the need for future studies on other indications.

## 1 Introduction

**FIGURE 1:**
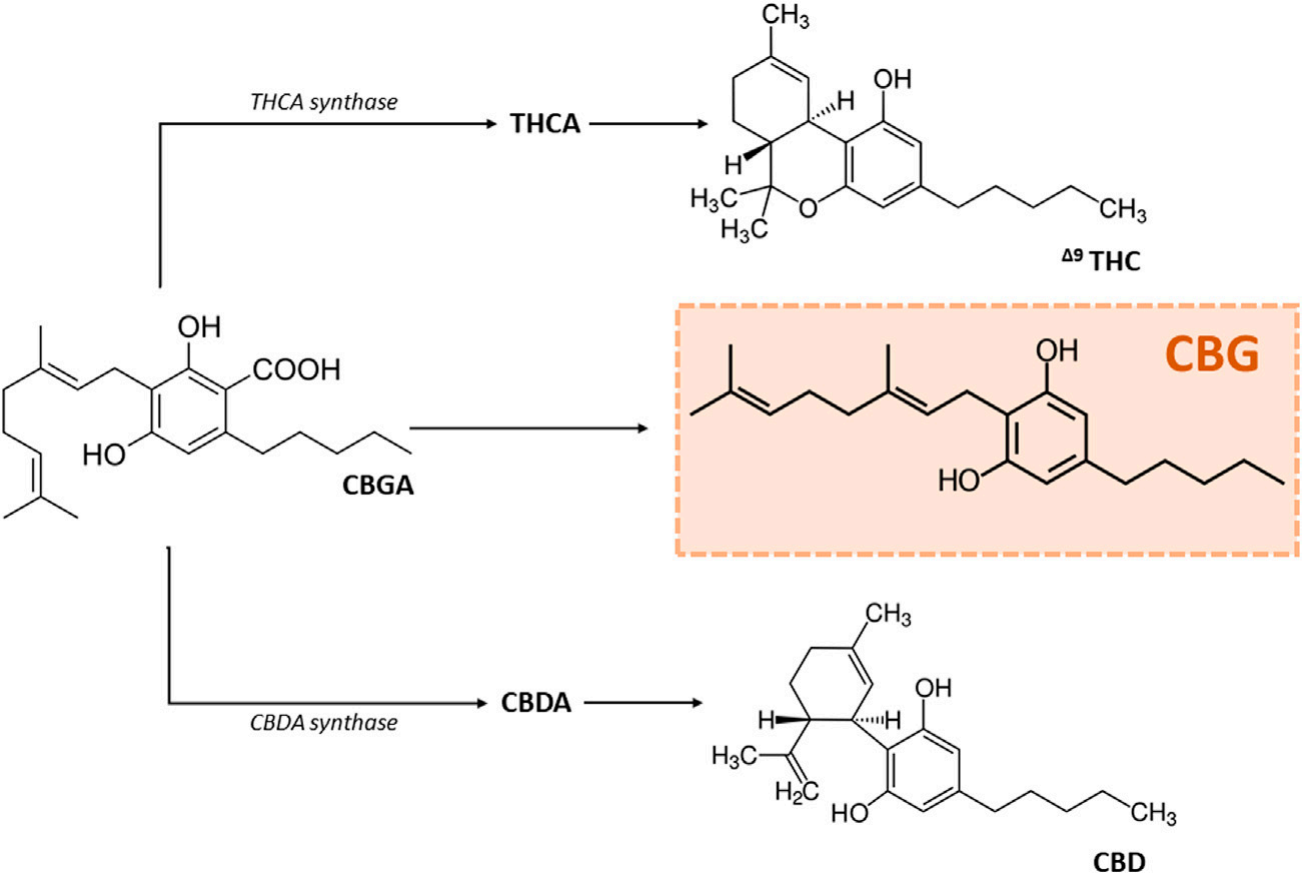
Scheme of the synthesis of the most popular phytocannabinoids. Abbreviations: CBD, cannabidiol; CBDA, cannabidiolic acid; CBG, cannabigerol; CBGA, cannabigerolic acid; Δ^9^-THC, Δ^9^-tetrahydrocannabinol; THCA, tetrahydrocannabidiolic acid.

Cannabinoids are chemical compounds that modulate a number of processes in the human body, mainly by interacting with cannabinoid receptors (CB-Rs). The current classification includes a) endocannabinoids [e.g., 2-arachidonoylglycerol (2-AG), N-arachidonoylethanolamine (anandamide; AEA)], b) phytocannabinoids isolated from *Cannabis* [cannabidiol (CBD), cannabigerol (CBG), Δ^9^-tetrahydrocannabinol (Δ^9^-THC)] and c) synthetic cannabinoids (e.g., WIN 55,212-2), Figure 1 (Kicman and Toczek, 2020; Krzyżewska et al., 2021; Maccarrone et al., 2023). The endocannabinoid system (ECS) have been shown to be widely distributed in the nervous, respiratory and cardiovascular systems, among others, and is involved in regulating its functions (Kicman et al., 2021; Remiszewski and Malinowska, 2022; Maccarrone et al., 2023).

**FIGURE 2:**
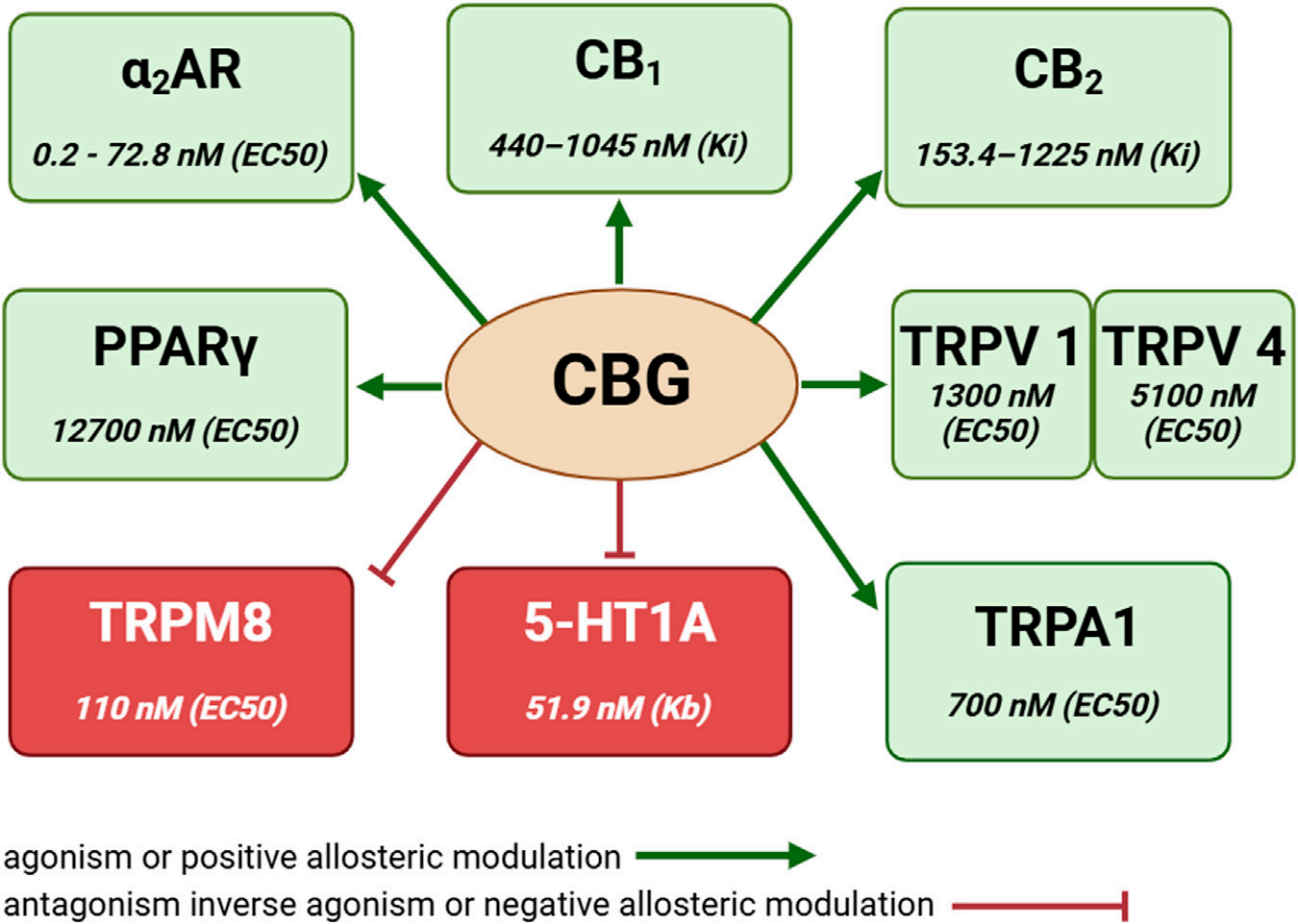
Cannabigerol and its receptor activity. Based on: Ryberg et al. (2007), Cascio et al. (2010), De Petrocellis et al. (2011), Granja et al. (2012), Borrelli et al. (2014), Navarro et al. (2018), Muller et al. (2019). Abbreviations: 5-HT_1A_, serotonin receptor type 1A; α_2_AR, alpha 2-adrenergic receptor; CB_1,2_, cannabinoid receptor type 1, 2; CBG, cannabigerol; EC50, half maximal effective concentration; Kb, equilibrium dissociation constant; Ki, inhibition constant; PPARγ, peroxisome proliferator-activated receptor gamma; TRPM8, transient receptor potential melastatin subfamily member 8; TRPA1, transient receptor potential ankyrin subfamily member 1; TRPV1,4, transient receptor potential vanilloid subfamily members 1, 4. Created with BioRender.

In the past years there has been an intense increase in interest in hemp products including the commercial use of CBG (Wilson-Poe et al., 2023). Cannabigerol is a non-psychoactive compound which exhibits unique properties not yet described for other cannabinoids, among them a potent alpha 2 adrenoceptor (α_2_AR) agonism (Cascio et al., 2010; Nachnani et al., 2021). However, unlike other well-studied phytocannabinoids (e.g.,: CBD or Δ^9^-THC) too little research has still been conducted on the therapeutic potential of CBG, and in particular on its effects on the cardiovascular system (Nachnani et al., 2021; Jastrząb et al., 2022). It has been reported that CBG exerts strong effects: a) antioxidant comparable to vitamin E, b) anti-inflammatory by reducing the activity of the central regulator of pro-inflammatory genes nuclear factor kappa-light-chain-enhancer of activated B cells (NF-κB), c) neuroprotective and neuromodulatory, d) antibacterial, and even e) anticancer potential (Valdeolivas et al., 2015; García et al., 2018; Dawidowicz et al., 2021; Jastrząb et al., 2022; Calapai et al., 2022; Aqawi et al., 2023; Li et al., 2024). Moreover, because CBG is a) an agonist of the α_2_AR, b) an agonist of the peroxisome proliferator-activated receptor gamma (PPARγ), and c) an antagonist of the serotonin receptor type 1A (5-HT_1A_), it has been speculated that it may have applications in the pharmacotherapy of the metabolic syndrome and its components, particularly hypertension and diabetes (Cascio et al., 2010; Nachnani et al., 2021; Jastrząb et al., 2022), see Figure 2.

In experiments on brain membranes, CBG has been shown to be the only currently known cannabinoid that is a potent α_2_AR agonist (EC_50_ = 0.2 nM; Cascio et al., 2010) and has the potential to reduce noradrenaline (NA) release from sympathetic nerve fibers, thereby alleviating vasoconstriction and lowering blood pressure (BP). This suggests that uncontrolled intake of CBG may result in unpredictable changes in BP, and may also interact with other cardiovascular drugs (Nachnani et al., 2021). Vernail et al. (2022) showed that a single intraperitoneal (*i.p.*) injection of CBG (3.3 and 10 mg/kg) to normotensive mice reduces mean blood pressure (MBP) in the manner sensitive to the α_2_AR antagonist - atipamezole. The same authors showed that CBG at a dose of 10 mg/kg in normotensive mice reduced BP and heart rate (HR) (Vernail et al., 2022). However, currently, there are no studies on how chronic CBG administration affects other cardiovascular parameters in hypertensive conditions, and the proposed mechanisms mediating the potential effect are only speculations.

Cardiovascular diseases, including hypertension, have been a leading cause of death worldwide for many years, and consistently elevated BP puts people at risk for serious cardiovascular incidents (stroke, heart attack). It is believed that primary hypertension, which accounts for 90% of cases, develops under the influence of a number of genetic and environmental factors (Mancia et al., 2023). According to the modified Page’s Mosaic Theory of Hypertension, overactivity of the sympathetic nervous system along with concomitant inflammation, increased oxidative stress and vascular endothelial dysfunction and many other (genetic factors, anatomical, environmental, endocrine, hemodynamic factors) are responsible for the progression of hypertension and many organ complications (Harrison et al., 2021; Remiszewski and Malinowska, 2022; McEvoy et al., 2024). Taking into account the previously mentioned beneficial effects of CBG, the purpose of our review was to analyse the therapeutic potential of CBG in cardiovascular diseases.

## 2 Materials and methods

To find articles on the potential cardiovascular effects of cannabigerol, PubMed and Web of Science (WoS) databases were searched. The time frame used was 1964-February 2025. To find precise information, each phrase was added to the term “cannabigerol,” respectively: “antioxidant,” “inflammation,” “cardiovascular,” “hypotensive,” “receptor affinity,” “adrenergic receptor,” “cannabinoid receptor,” “PPAR,” “clinical trials,” “TRPA1,” “insulin resistance,” “hemostasis,” “animal studies,” and “fibrosis.” During the search, the phrase “cannabigerol” was combined with only one keyword. Titles were analyzed first, followed by abstracts and full texts of articles. Exclusion criteria included articles in a language other than English, articles without full access, duplicates, articles where cannabigerol was only marginally mentioned, studies not addressing the main issue, and studies measuring other indicators like the antibacterial effect of cannabigerol. The types of articles considered were full-text research articles. Review papers were used as a general summary but not as the main source of data. Editorial comments, letters to the editor, articles without scientific review, and conference abstracts were not included in the review.

## 3 Results

Table 1 shows the results of the search described in the materials and methods section. After applying the exclusion criteria described in the methods, 34 papers were used to prepare the section on cannabigerol. The other papers were used as a general background to the topic. One exception was made for the conference abstract - Vernail et al. (2023). Chronic cannabigerol administration lowers blood pressure in phenotypically normal mice. Physiology. 38. https://doi.org/10.1152/physiol.2023.38.S1.5726031, which we considered relevant in the context of our review.

**TABLE 1:** Records identified from databases.

Keywords	Database	
	PubMed	WoS
cannabigerol	515	518
cannabigerol + antioxidant	46	41
cannabigerol + inflammation	53	61
cannabigerol + cardiovascular	6	6
cannabigerol + hypotensive	3	2
cannabigerol + receptor affinity	14	18
cannabigerol + PPAR	6	6
cannabigerol + clinical trials	23	53
cannabigerol + TRPA1	7	10
cannabigerol + insulin resistance	3	3
cannabigerol + hemostasis	1	-
cannabigerol + animal studies	84	28
cannabigerol + fibrosis	2	3

Abbreviations: PPAR, peroxisome proliferator-activated receptor; TRPA1, transient receptor potential A1; WoS, web of science.

## 4 Discussion

### 4.1 Cannabis in cardiovascular diseases

The use of *Cannabis sativa* for recreational purposes and all kinds of ailments such as pain or digestive disorders dates back thousands of years. The growing interest in cannabis products and medical marijuana has resulted in many scientific publications on the therapeutic potential of cannabinoids and has contributed to the introduction of several well-known cannabis-based drugs to the pharmaceutical market e.g., Sativex, Epidiolex and others (Legare et al., 2022; Wechsler et al., 2024). *Cannabis sativa L.* var. *Indica* plant includes about 700 compounds, more than 100 of which are cannabinoids, such as Δ^9^-THC, CBD, CBG and many others (Di, 2006; Remiszewski and Malinowska, 2022; Weresa et al., 2022; Oriola et al., 2024). Expression of ECS components was found in the cardiovascular system, suggesting that they may be involved in regulating its function (Remiszewski and Malinowska, 2022; Weresa et al., 2022). Some cannabinoids (e.g., CBD, AEA, 2-AG) exhibit remarkable pulmonary and systemic vasorelaxant properties, antioxidant and anti-inflammatory effects (e.g., CBD, CBG), which may make them attractive therapeutic targets for treating systemic and pulmonary hypertension (PH) (Baranowska-Kuczko et al., 2020; Krzyżewska et al., 2021). However, despite emerging reports that acute intravenously (*i.v.*) administration of certain cannabinoids (AEA, methanandamide (MethAEA) and HU210) lowers BP in spontaneously hypertensive rats (SHRs) (stronger than in normotensive Wistar Kyoto (WKY) rats) (Li et al., 2003; Bátkai et al., 2004; Godlewski et al., 2010; Malinowska et al., 2019), studies involving chronic administration of cannabinoids to hypertensive rats confirmed that only endocannabinoid-like molecule - palmitoylethanolamide (PEA) lowered BP in SHRs after 5 weeks of subcutaneous (*s.c.*) administration (Mattace Raso et al., 2015; Remiszewski and Malinowska, 2022). However, despite that chronic CBD administration does not show hypotensive effects in rats with primary and secondary hypertension (Remiszewski et al., 2020), the same dose of CBD (10 mg/kg) has been shown to attenuate monocrotaline-induced PH in the rat and Sugen hypoxia-induced PH in mice by lowering right ventricular systolic blood pressure (Sadowska et al., 2020; Lu et al., 2021).

### 4.2 The role of α_2_AR in hypertension

Cannabigerol is a highly potent α_2_AR agonist. Cascio et al. (2010) showed that CBG inhibits the electrically induced contractions of the vas deferens in the manner sensitive to the α_2_AR antagonist (yohimbine). Importantly, CBG produces this effect with the potency which is similar to the potency of the well-known α_2_AR agonists clonidine and dexmedetomidine in the same bioassay (Cascio et al., 2010). α_2_-adrenergic receptors (consisting of α_2A_, α_2B_ and α_2C_ subtypes) are Gi-coupled G-protein coupled receptors (GPCRs) and are located in the cardiovascular system, kidneys (which affects BP regulation), as well as in platelets and the brain (Proudman et al., 2022). Antihypertensive drugs, which are α_2_AR agonists, are designed to activate these receptors to reduce BP (Proudman et al., 2022). Activation of presynaptic α_2_AR leads to inhibition of NA release thereby reducing activity of sympathetic neurons innervating the heart and blood vessels (Proudman et al., 2022). One of the most popular hypotensive drug belonging to the α_2_AR and imidazole receptor agonist group is clonidine (National Institute of Diabetes and Digestive and Kidney Diseases, 2017; Srivastava et al., 2020). Clonidine interacts with the α_2_ARs at both peripheral, central, presynaptic and postsynaptic levels. Probably due to this fact clonidine can cause side effects, and currently, when considering agonists of the α_2_AR, drugs or therapies targeted at one subtype of the α_2_AR are sought, which will limit side effects and use the maximum receptor potential. That is why it is so important to determine the exact mechanisms of action of the new α_2_AR agonists (Manzon et al., 2023).

**FIGURE 3:**
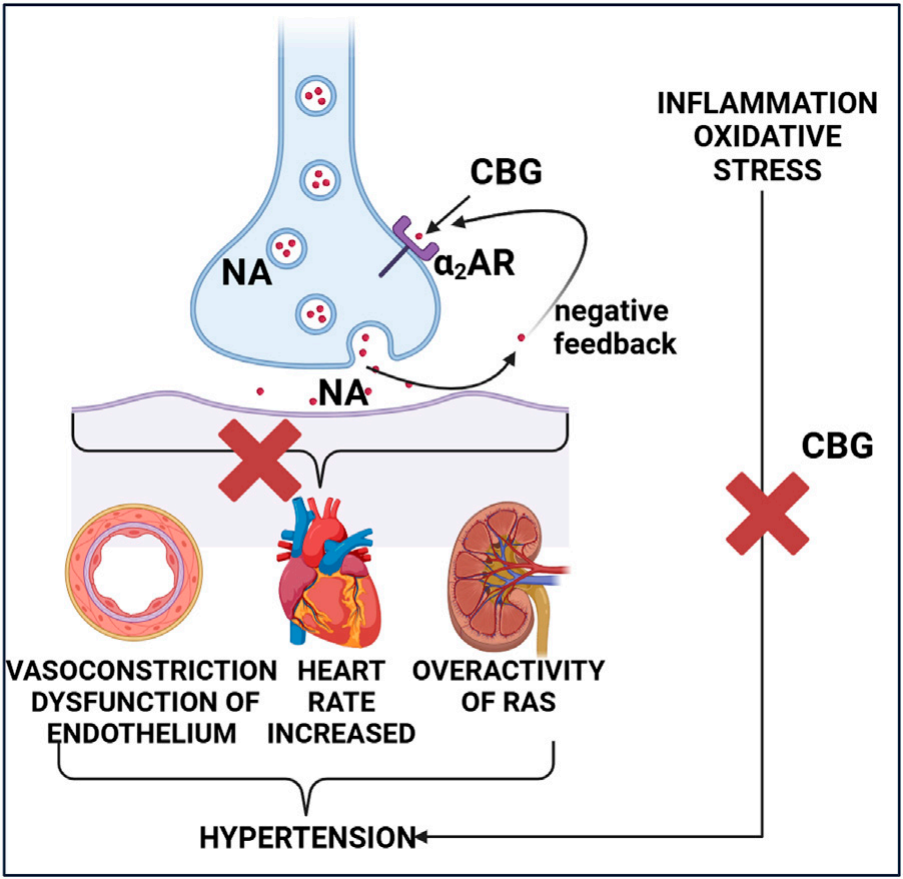
The potential antihypertensive effect of cannabigerol. Abbreviations, α_2_AR, alpha 2 adrenoceptor; CBG, cannabigerol; NA, noradrenaline; RAS, renin-angiotensin system. Created with BioRender.

The sympathetic nerve activity is increased in hypertensive patients which leads to enlarged NA release. Presynaptic α_2_ARs (acting as autoreceptors) control sympathetic neurotransmission in through a negative feedback mechanism (Figure 3) (Hering et al., 2020a). Pharmacological blockade or genetic deletion of α_2_ARs accelerates hypertension and kidney damage through multiple mechanisms, including the impaired negative feedback, resulting in an increased amount of NA in the end-organs (Schmieder, 2010; Hering et al., 2020b).

Vernail et al. (2022) showed that a single administration of CBG at doses of 3.3 mg/kg and 10 mg/kg (*i.p*.) reduces MBP in normotensive mice by −22 ± 2 and −28 ± 2 mmHg from baseline values, respectively. This effect is probably mediated by α_2_ARs because the use of the antagonist - atipamezole (3 mg/kg, *i. p*.) abolished the depressant effect of CBG. In addition, CBG showed a lower hypotensive effect than guanfacine (1 mg/kg, *i. p*.), which is a selective central α_2A_AR subtype agonist. Therefore, the authors speculate that this effect may be due to the fact that CBG: is a weaker α_2_AR agonist than guanfacine and/or involves a different α_2_AR subtype, and/or its potential cardiovascular effects may result from peripheral and central effects on α_2_ARs (Vernail et al., 2022). The same group of researchers showed that chronic administration of CBG for 14 days at a dose of 10 mg/kg lowers systolic blood pressure (SBP) and HR but the mechanism of action remains unclear (Vernail et al., 2023). As mentioned, there are three subtypes of α_2_ARs. It is hypothesized that the α_2A_ARs subtypes are predominantly located presynaptically and act as autoreceptors for NA, thereby causing BP lowering, while the α_2B_ARs predominate on the postsynaptic membranes and may be responsible for the transient initial hypertensive effect and for vasoconstriction of oral α_2_AR agonists (Philipp et al., 2002; Maaliki et al., 2019). The same authors observed no changes in the HR parameter after CBG (10 mg/kg) administration in mice (Vernail et al., 2022). It is worth noting that mice have a higher resting HR than rats, thus it cannot be ruled out that CBG may cause an effect on HR in species with lower resting HR (Weresa et al., 2022). In the context of hypertension, it is also worth mentioning that Fleisher-Berkovich et al. (2023) showed that telmisartan (a known antihypertensive drug), exhibited an additive effect with CBG, resulting in inhibition of nitric oxide (NO) production in lipopolysaccharide (LPS)-stimulated microglia. However, it is known that reducing NO in the cardiovascular system can induce vasoconstriction and increase BP (Bryan, 2022) so it is lucrative to determine the precise mechanism of action of this interaction.

### 4.3 Influence of cannabigerol on oxidative stress

A number of studies indicate a link between oxidative mechanisms and the overproduction of reactive oxygen species (ROS) and the development of hypertension (Araujo and Wilcox, 2014; Lopes et al., 2015; Camargo et al., 2018; Vaka et al., 2020; Franco et al., 2022). It is known that redox imbalance accelerates vascular aging and reduces the bioavailability of NO (Bachschmid et al., 2013; Tracy et al., 2021). The consequence of the above changes is increased vascular stiffness, impaired vascular relaxation and vascular endothelial dysfunction, which can exacerbate the progression of hypertension (Korsager Larsen and Matchkov, 2016; Guzik and Touyz, 2017).

Studies show that CBG may be a promising agent for the adjunctive treatment of oxidative stress-related diseases (Giacoppo et al., 2017; Calapai et al., 2022; Fleisher-Berkovich et al., 2023) and its antioxidant effect is comparable to that of vitamin E (Dawidowicz et al., 2021). Giacoppo et al. (2017) showed that CBG reduces oxidative stress in hydrogen peroxide (H_2_O_2_)-stimulated macrophages, and this effect was attenuated after administration of a cannabinoid type 2 receptor (CB_2_-R) antagonist (AM630), suggesting that CBG regulates oxidative stress through interaction with these receptors. This is consistent with reports that CB_2_-R activation exhibits antioxidant and anti-inflammatory effects (Kumawat and Kaur, 2019). The beneficial antioxidant properties of CBG include inhibition of inducible nitric oxide synthase (iNOS), nitrotyrosine and Poly (ADP-ribose) polymerase (PARP-1), modulation of mitogen-activated protein kinase, and reduction of NF-κB transcriptional activity. In addition, by regulating the expression of superoxide dismutase-1 (SOD-1), CBG enhances cellular antioxidant defence capabilities and inhibits apoptosis (Giacoppo et al., 2017; Fleisher-Berkovich et al., 2023) (Table 2).

**TABLE 2:** A summary of the so far known properties of cannabigerol in *vitro* studies on cell lines.

Cell line	Concentration of CBG	Effect	Proposed mechanism of action	References
RAW 264.7 cells treated with H_2_O_2_	10 μM	Anti-inflammatory, antioxidant: ↓ JNK, NF-κB, nitrotyrosine, iNOS, PARP-1 ↑ IκB-α, SOD-1 Regulation of apoptosis: ↓ Bax ↑ Bcl-2	effects mediated by CB_2_ receptors; confirmed the lack of involvement of CB_1_ receptors	Giacoppo et al. (2017)
BV2 microglia treated with LPS	5 µM	Anti-inflammatory, antioxidant: ↓ NO, TNF-α, iNOS	-	Fleisher-Berkovich et al. (2023)
	10 µM	Anti-inflammatory antioxidant: ↓ NO, iNOS	-	
NSC-34 cells treated with the medium of LPS-stimulated macrophages	7.5 µM	Anti-inflammatory, antioxidant: ↓ nitrotyrosine, iNOS, SOD-1, IL-1β, TNF-α, IFN-γ ↑ Nrf-2 Regulation of apoptosis: ↓ cleaved caspase 3, Bax ↑ Bcl-2	-	Gugliandolo et al. (2018)
cell medium of murine peritoneal macrophages treated with LPS	0.001 μM	↔ nitrates	confirmed the lack of involvement of CB_1_ receptors; probable involvement of CB_2_ receptors	Borrelli et al. (2013)
	0.01 μM	Antioxidant: ↓ nitrates		
	0.1 μM	Antioxidant: ↓ nitrates		
	1 μM	Antioxidant: ↓ iNOS (protein level), nitrates ↔ iNOS (mRNA expression)		
Ptk6 null colonic epithelial cells exposed to H_2_O_2_/Fe^2+^	0.1 μM	↔ ROS production	-	
	1 μM	Antioxidant: ↓ ROS production		
	10 μM	Antioxidant: ↓ ROS production		

Abbreviations: Bax, Bcl-2-associated X protein; Bcl-2, B-cell lymphoma 2; CB_1,2_, cannabinoid receptor type 1, 2; Fe^2+^, ferrous ion; H_2_O_2_, hydrogen peroxide; IκB-α, inhibitor of nuclear factor kappa B; IL-1β, interleukin 1 beta; IFN-γ, interferon gamma; iNOS: nitric oxide synthase; JNK, c-Jun amino-terminal kinase; LPS, lipopolysaccharide; NF-κB, nuclear factor kappa-light-chain-enhancer of activated B cells; NO, nitric oxide; Nrf-2, nuclear factor erythroid 2-related factor 2; NSC-34, motor neuron-like hybrid cell line, Neural Stem Cells 34; PARP-1, poly (ADP-ribose) polymerase-1; Ptk6, protein tyrosine kinase 6; ROS, reactive oxygen species; SOD-1, superoxide dismutase 1; TNF-α, tumour necrosis factor alpha.

Although CBG is generally considered to be a compound with beneficial antioxidant effects, a recent study showed that CBG administration (1.33 mg/kg/day) to rats for 90 days resulted in increased concentrations of malondialdehyde (MDA), which is a product of lipid peroxidation, carbonylated proteins, and led to an increase in total oxidative stress and a decrease in total antioxidant activity in the plasma and/or liver of rats (Table 3) (Polanska et al., 2023). However, it should also be kept in mind that a similar trend was also observed with CBD, which is considered to be generally safe and well tolerated where chronic administration of CBD (10 mg/kg/day) for 2 weeks increased levels of plasma lipid peroxidation markers MDA, 4-hydroxynonenal (4-HNE) and 4-hydroxyhexenal (4-HHE) in healthy rats, but this was not observed in hypertensive rats (SHR) (Remiszewski et al., 2020). Given the growing interest in the use of cannabis products in pharmacotherapy, these discrepancies require further extended research, especially attempts to explain the reasons for such different effects of cannabinoids. One potential explanation for this phenomenon could be the biphasic effects of cannabinoids, which means that their effects can be different or even opposite depending on the dose. Among other things, the biphasic effects of cannabinoids affect the modulation of motor activity, anxiety reactions or motivational processes (Shustorovich et al., 2024). Christie et al. (2020) showed that low doses (1–10 nM) of methanandamide decreased the stretch responses of the afferent fibers of the gastric vagus nerve, while high doses (30–100 nM) increased this response. Another study found that 0,1 mg/kg of Δ9-THC induced hyperactivity, while 1 mg/kg induced hypoactivity in rats (Katsidoni et al., 2013). Interestingly, Δ9-THC at a concentration of 0.08 μM improved the survival of zebrafish (*Danio rerio*), but higher concentrations of THC (2 μM) prevented this effect. Low concentrations of THC (0.08 μM), as opposed to higher concentrations (2 μM), improved fertility, and reduced the expression of pro-inflammatory cytokines including tumor necrosis factor alpha (TNF-α), interleukin 6 (IL-6) and interleukin 1 beta (IL-1β) in the liver (Pandelides et al., 2020). It is speculated that activation of cannabinoid receptors, as well as regulation of the gamma-aminobutyric acid (GABA)/glutamate neurotransmitter balance by cannabinoids may be responsible for their biphasic effects, however, further molecular studies are needed for precise dosing that achieves the desired therapeutic effect while minimizing side effects (Rey et al., 2012).

**TABLE 3:** A summary of the so far known properties of cannabigerol in *in vivo* experimental studies under physiological and pathological conditions.

Species	Route	Dose	Material	Effect	References
Physiological conditions					
male mice	*i.p*.	3.3 mg/kg (once)	—	Hypotensive:	Vernail et al. (2022)
				↓ MBP, SBP, DBP, HR	
				↔ locomotor activity	
		10 mg/kg (once)	—	Hypotensive:	
				↓ MBP, SBP, DBP	
				↔ HR, locomotor activity	
male mice	*i.p.*	10 mg/kg/day for 14 days	—	Hypotensive:	Vernail et al. (2023)
				↓ MBP, SBP, DBP, HR	
				↔ locomotor activity	
male rats	*i.g.*	0.66 mg/kg/day for 90 days	—	↓ body weight, liver weight, liver/body weight ratio	Polanska et al. (2023)
			blood/plasma	↓ WBC, MONO, LYMPH (%), PLT, RDW-SD, RDV-CV, PDW, PCT, BASO,	
				ALT, LDH, AMYL2,CREA, K^+^, CA^2+^	
				↑ MCH, MCHC, NEUT (%)	
				↔ RBC, HGB, HCT, MCV, MPV, P-LCR, NRBC, NEUT, EO, IG, BASO (%), ALB, AST, ALP, BILT, GGT, GLU, TP, TRIGL, UA, UREA, Na^+^, Cl^-^	
				Pro-oxidant:	
				↓ antioxidant capacity	
				↔ MDA, carbonyl proteins, oxidative stress	
			liver	Pro-oxidant:	
				↑ carbonyl proteins	
				↔ MDA, oxidative stress, antioxidant capacity	
		1.33 mg/kg/day for 90 days	—	↓ body weight, liver weight, liver/body weight ratio	
			blood/plasma	↓ WBC, MONO, LYMPH (%), MONO (%), BASO (%), PLT, RDW-SD, RDV-CV, PCT, LYMPH, BASO,	
				ALT, LDH, TRIGL, CREA, CA^2+^, Na^+^, K^+^	
				↑ MCH, MCHC, NEUT (%), EO (%)	
				↔ RBC, HGB, HCT, MCV, PDW, MPV, P-LCR, NRBC, NEUT, EO, IG, ALB, AST, ALP, AMYL2, BILT, GGT, GLU, TP, UA, UREA, Cl- Pro-oxidant:	
				↓ antioxidant capacity	
				↑ MDA, oxidative stress	
				↔ carbonyl proteins	
			liver	Pro-oxidant:	
				↓ antioxidant capacity	
				↑ MDA, oxidative stress, carbonyl proteins	
male mice	*i.p.*	2.46 mg/kg/ 3 times a week for 2 weeks	—	↔ food consumption, body weight, liver/body weight ratio	Aljobaily et al. (2022)
			liver	↔ CD36, TRIGL, CD45, F4/80 (mRNA expression and immunofluorescence staining), fibrosis, α-SMA (mRNA expression and immunofluorescence staining), CB_1_, CB_2_	
		24.6 mg/kg/ 3 times a week for 2 weeks	—	↔ food consumption, body weight, liver/body weight ratio	
			liver	↔ CD36, TRIGL, F4/80 (mRNA expression), α-SMA (mRNA expression and immunofluorescence staining), CB_1_, CB_2_	
				Pro-inflammatory:	
				↑ CD45, F4/80 (immunofluorescence staining)	
				Pro-fibrotic:	
				↑ fibrosis	
male rats	*i.g.*	30 mg/kg for 14 days	plasma	Modulate of lipid metabolism:	Bzdęga et al. (2023)
				↑ SFA, SFA1P	
				↔ SFO, S1P, CER, SPH	
			liver	Modulate of lipid metabolism:	
				↓ SFO, CER, CerS5	
				↑ SFA, S1P, SFA1P, ASAH2 ↔ SPH, SPTLC1, SPTLC2, CerS2, CerS4, CerS6, ASAH1, ASAH3, SPHK1, SPHK2, AlK-SMase, N-SMase, S1PR2, S1PR3, SGPL1, CERT, SPNS2, ABCA1 Modulate insulin signaling pathway: ↓ pGSK-3β Ser 9/GSK-3β, pGSK-3α Ser 21/ GSK-3β, pGSK-3α Tyr 279/GSK-3α, pGSK-3β Tyr 216/GSK-3β ↑ pAkt Ser 472, 473, and 474/Akt ↔ pAkt Thr 308, 309, and 305/Akt Modulate insulin sensitivity and body weight: ↓ glycogen, ↔ body weight, Insulin Tolerance Test Modulate expression of proteins associated with fatty acids and glucose metabolism: ↓ FAS, ↑ ACC2	
				↔ SREBP-1c (precursor and mature), pACC2 Ser 9, PDH	
male rats	*i.g.*	30 mg/kg for 14 days	muscle	Modulate of lipid metabolism:	Bielawiec et al. (2024)
				↓ PS and PI n3 activity pathway, total PL n6 activity pathway	
				↑ total PL, PE, PI; SCD1 activity in total PE; PC and PE n3 activity pathway	
				↔ PC, PS; SCD1 activity in total PL, PC, PS and PI; SCD1, ELOVL3, 5 and 6, FAD S1 and S2; total PL n3 activity pathway; PC, PS, PI and PE n6 activity pathway	
				Inflammation:	
				↔ cPLA2, COX-1, COX-2, 5-LOX, 12/15 LOX, PPARγ, NF-κB, Nrf-2	
				Remodelling and fibrosis:	
				↔ MMP-2, MMP-9, collagen 1a and 3a	
male rats	*i.g.*	30 mg/kg/day for 14 days	colon	Modulate of lipid metabolism:	Sztolsztener et al. (2024)
				↓ AA content in TAG ↑ n-3 PUFA pathway activity in PL, TAG, DAG and FFA; n-6 PUFA pathway activity in FFA ↔ n-6/n-3 PUFA ratio in PL, DAG, TAG and FFA; n-6 PUFA pathway activity in PL, DAG and TAG; AA content in PL, DAG and FFA Inflammation: ↑ NF-κB, Nfr-2 ↔ 5-LOX, 12/15-LOX, PGE2, PGI2, IL-6, cPLA_2,_ COX-1, COX-2, LTC4, LTB4, LXA4 Remodelling and fibrosis: ↔ TGF-β , MMP-2, MMP-9, collagen 1a and 3a	
male rats	*p.o.*	30 mg/kg once	—	↔ food intake, locomotor activity	Brierley et al. (2016)
		60 mg/kg once		↔ food intake, locomotor activity	
		120 mg/kg once		↑ food intake ↔ locomotor activity	
		240 mg/kg once		↑ food intake, locomotor activity	
pathological conditions					
male mice with non-alcoholic steatohepatitis	*i.p.*	2.46 mg/kg/ 3 times a week for 2 weeks	—	↓ liver/body weight ratio ↔ food consumption, body weight	Aljobaily et al. (2022)
			liver	↓ CB_1_, CB_2_ ↔ CD36, TRIGL, F4/80 (mRNA expression and immunofluorescence staining) Anti-inflammatory: ↓ CD45 Anti-fibrotic: ↓ fibrosis, α-SMA (immunofluorescence staining), α-SMA (mRNA expression)	
		24.6 mg/kg/3 times a week for 2 weeks	—	↔ food consumption, body weight, liver/body weight ratio	
			liver	↔ CD36, TRIGL, CB_1_, CB_2_ Pro-inflammatory: ↑ CD45, F4/80 (mRNA expression and immunofluorescence staining) Remodeling and fibrosis: ↓ α-SMA (immunofluorescence staining) ↔ fibrosis, α-SMA (mRNA expression)	
male mice with experimental colitis	*i.p.*	Preventive protocol: 1, 5 and 30 mg/kg once a day for six consecutive days starting 3 days before DNBS administration	—	↓ colon weight/length ratio	Borrelli et al. (2013)
		Treatment protocol: 1 mg/kg for two consecutive days starting 24-h after DNBS administration	—	↔ colon weight/length ratio	
		Treatment protocol: 5 mg/kg for two consecutive days starting 24-h after DNBS administration	—	↓ colon weight/length ratio	
		Treatment protocol: 30 mg/kg for two consecutive days starting 24-h after DNBS administration	—	↓ colon weight/length ratio	
			blood	↓ intestinal permeability	
			colon	↔ COX-2 Anti-inflammatory, antioxidant: ↓ MPO, IL-1β, IFN-γ, iNOS ↑ IL-10, SOD-1	
male rats with obese and insulin resistance	*i.g.*	30 mg/kg/day for 14 days	plasma	Modulate of lipid metabolism: ↓ SFA1P, SP1 ↑ CER ↔ SFO, SFA, SPH	Bzdęga et al. (2023)
			liver	Modulate of lipid metabolism: ↓ SFA1P, CerS5, N-SMase, S1PR2, ABCA1 ↑ SFA, S1P, SPH, CerS6, ASAH3, SPHK1 ↔ SFO, CER, SPTLC1, SPTLC2, CerS2, CerS4, ASAH1, ASAH2, SPHK2, AlK-SMase, S1PR3, SGPL1, CERT, SPNS2 Modulate insulin signaling pathway: ↓ pGSK-3β Tyr 216/GSK-3β ↑ pAkt Ser 472, 473, and 474/Akt; pAkt Thr 308, 309, and 305/Akt ↔ pGSK-3β Ser 9/GSK-3β; pGSK-3α Ser 21/ GSK-3β; pGSK-3α Tyr 279/GSK-3α Modulate insulin sensitivity and body weight: ↓ body weight, glycogen ↔ Insulin Tolerance Test Modulate expression of proteins associated with fatty acids and glucose metabolism: ↓ FAS, SREBP-1c mature, ACC2, pACC2 Ser 9 ↔ SREBP-1c precursor, PDH	
male rats with obese and insulin resistance	*i.g.*	30 mg/kg/day for 14 days	muscle	Modulate of lipid metabolism: ↓ PC, PS, SCD1 activity in total PL and PS; SCD1, ELOVL3 and 6, FAD S1 and S2, PS n3 activity pathway; PC and PI n6 activity pathway ↑PE, PI; SCD1 activity in PC and PI; ELOVL5 PC, PI and PE n3 activity pathway ↔ total PL; SCD1 activity in PE; total PL n3 activity pathway; total PL, PS and PE n6 activity pathway Anti-inflammatory: ↓ cPLA2, COX-1, COX-2, 5-LOX, 12/15 LOX, NF-κB ↑ PPARγ, Nrf-2 Remodelling and fibrosis: ↑ MMP-2, MMP-9, collagen 1a, collagen 3a	Bielawiec et al. (2024)
male rats with obese and insulin resistance	*i.g.*	30 mg/kg/day for 14 days	colon	Modulate of lipid metabolism: ↓ n-6/n-3 PUFA ratio in TAG; AA content in PL, TAG ↑ n-3 and n-6 PUFA pathway activity in TAG; n-3 PUFA pathway activity in DAG and FFA ↔ n-6/n-3 PUFA ratio in PL, DAG and FFA; n-3 and n-6 PUFA pathway activity in PL; n-6 PUFA pathway activity in DAG and FFA; AA content in DAG and FFA Anti-inflammatory: ↓ cPLA_2,_ COX-1, COX-2, 12/15-LOX, LTB4, NF-κB ↑ LXA4, Nfr-2 ↔ 5-LOX, PGE2, PGI2, LTC4, IL-6 Remodelling and fibrosis: ↓ TGF-β, collagen 3a ↔ MMP-2, MMP-9, collagen 1a	Sztolsztener et al. (2024)

Abbreviations: 5-LOX and 12/15-LOX, 5- and 12/15-lipoxygenase; α-SMA, alpha smooth muscle actin; AA, arachidonic acid; ABCA1, ATP-binding cassette transporter; ACC2, acetyl-CoA, carboxylase 2; Akt, protein kinase B; ALB, albumin; AlK-SMase, alkaline sphingomyelinase; ALP, alkaline phosphatase; ALT, alanine transaminase; AMYL2, α-amylase; ASAH1, acid ceramidase; ASAH2, neutral ceramidase; ASAH3, alkaline ceramidase; AST, aspartate transaminase; BASO, basophils; BILT, total bilirubin; CA2, ionized calcium; CB_1,2_, cannabinoid receptor type 1, 2; CD36, cluster of differentiation 36; CD45, cluster of differentiation 45; CER, ceramide; CerS2, dihydroceramide synthase 2; CerS4, dihydroceramide synthase 4; CerS5 dihydroceramide synthase 5; CerS6, dihydroceramide synthase 6; CERT, ceramide transport protein; Cl^−^, chlorides; COX-1/2 cyclooxygenase 1 and 2; cPLA_2_, cytosolic phospholipase A2; CREA, creatinine; DAG, diacylglycerol; DBP, diastolic blood pressure; ELOVL3, ELOVL5, and ELOVL6, fatty acid elongase 3, 5 and 6; EO, eosinophils; FADS1/2, fatty acid desaturase 1 and 2; FAS, fatty acid synthase; FFA, free fatty acid; GGT, gammaglutamyl transferase; GLU, glucose; GSK-3α/β, glycogen synthase kinase B-3alpha/beta; HGB, haemoglobin; HCT, haematocrit; HR: heart rate; *i.g*., intragastric administration; IG, immature granulocytes; IL-1/6/10, interleukin 1/6/10; IFN-γ, interferon gamma; iNOS, inducible nitric oxide synthase; *i.p*.: intraperitoneal administration; K^+^, potassium; LDH, lactate dehydrogenase; LTB4, leukotriene B4; LTC4, leukotriene C4; LXA4, lipoxin A4; LYMPH, lymphocytes; MBP, mean blood pressure; MCH, mean cell haemoglobin; MCHC, mean corpuscular haemoglobin concentration; MCV, mean corpuscular volume; MDA, malondialdehyde; MMP-2/9 matrix metalloproteinases 2/ 9; MONO, monocytes; MPO, myeloperoxidase; MPV, mean platelet volume; N-SMase, neutral sphingomyelinase; Na^+^, sodium; NEUT, neutrophils; NF-κB, nuclear factor kappa-light-chain-enhancer of activated B cells; NRBC, nucleated red blood cell; Nrf-2, nuclear factor erythroid 2-related factor 2; P-LCR, platelet larger cell ratio; pACC2 Ser 9, phosphorylated acetyl-CoA, carboxylase 2 Ser 9; pAkt Thr 308, 309, and 305: phosphorylated protein kinase B in Thr 308, 309, and 305; pAkt Ser 472, 473, and 474, phosphorylated protein kinase B in Ser 472, 473, and 474; PC, phosphatidylcholine; PCT, percentage volume occupied by platelets; PDH, pyruvate dehydrogenase; PDW, platelet distribution width; PE, phosphatidylethanolamine; PGE2, prostaglandin E2; PGI2, prostacyclin I2; PI, phosphatidylinositol; PL, phospholipid fraction; PLT, platelet count; *p.o*., per os administration; PPARγ, peroxisome proliferator-activated receptor gamma; PS, phosphatidylserine; PUFAs, polyunsaturated fatty acids; RBC, red blood cells; RDW-SD, red blood distribution width-standard deviation; RDV-CV, red blood cell distribution width-variation coefficient; S1P, sphingosine-1-phosphate; S1PR2, sphingosine-1-phosphate receptor 2; S1PR3, sphingosine-1-phosphate receptor 3; SBP, systolic blood pressure; SCD1, stearoyl-coenzyme A desaturase 1; SFA, sphinganine; SFA1P, sphinganine-1-phosphate; SFO, sphingosine; SGPL1, sphingosine-1-phosphate lyase 1; SOD-1, superoxide dismutase-1; SPH, sphingomyelin; SPHK1, sphingosine kinase 1; SPHK2, sphingosine kinase 2; SPNS2, sphingolipid transporter 2; SPTLC1, serine palmitoyltransferase 1; SPTLC2, serine palmitoyltransferase 2; SREBP-1c, sterol regulatory element-binding protein-1c precursor; TAG, triacylglycerol; TGF-β, transforming growth factor beta; TP, total proteins; TRIGL, triglycerides; UA, uric acid; UREA, urea; WBC, white blood cells.

### 4.4 Influence of cannabigerol on inflammation

A systemic inflammatory response accompanies the development of hypertension, and promotes dysfunction of blood vessels, kidneys, and other end-organs which further exacerbates the increase in BP acting as a positive feedback loop (Xiao and Harrison, 2020). Studies show that hypertensive patients have increased levels of inflammatory markers such as C-reactive protein, TNF-α, IL-6, IL-1β, interleukin 18 (IL-18) and also monocyte chemoattractant protein 1 (MCP-1) (Dalekos et al., 1997; Madej et al., 2005; Rabkin, 2009; Schnabel et al., 2008; Thomas et al., 2021). Numerous experiments on animal models show that targeting the aforementioned points and many other immune pathways has beneficial effects such as: lowering BP, reducing vascular inflammation, inhibiting kidney damage or inhibiting cardiac hypertrophy and dysfunction (Murray et al., 2021; Veiras et al., 2021; Thomas et al., 2021; Veiras et al., 2022; Zhang et al., 2023).

Currently, data on CBG’s effects on inflammation in the cardiovascular system are lacking, but there are indications that CBG has anti-inflammatory potential. Aljobaily et al. (2022) showed that a low dose of CBG (2.46 mg/kg/day) reduced leukocyte infiltration, particularly of macrophages in the liver of mice with non-alcoholic steatohepatitis, while a high dose (24.6 mg/kg/day) was not effective. Pre-treatment with CBG (7.5 µM) reduced levels of the pro-inflammatory cytokines IL-1β, TNF-α and interferon gamma (IFN-γ) in motor neuron-like hybrid cell line (Neural Stem Cells 34 -NSC-34) treated with LPS-stimulated macrophage medium (Gugliandolo et al., 2018) confirming that CBG is able to modulate important inflammatory pathways involved in the pathogenesis of cardiovascular disease (Table 2). Other authors showed that in a mouse model of bowel disease, treatment with CBG (30 mg/kg/day) reduced levels of pro-inflammatory cytokines: IL-1β and IFN-γ, and increased levels of anti-inflammatory interleukin 10 (IL-10) in the colon, suggesting the usefulness of CBG in the treatment of typically inflammatory diseases (Borrelli et al., 2013) (Table 3). Other potentially beneficial anti-inflammatory effects of CBG include decreasing NF-κβ inhibitor alpha (Iκβ-α) phosphorylation, which inhibits the major regulator of pro-inflammatory genes-NF-κB, as well as decreasing cyclooxygenase 1 and 2 (COX-1 and COX-2) activity (Ruhaak et al., 2011; Shah et al., 2013; Giacoppo et al., 2017; Jastrząb et al., 2022). Moreover, CBG was effective in inhibiting TNF-α-induced production of IL-6 and interleukin 8 (IL-8) by rheumatoid synovial fibroblasts (Lowin et al., 2023).

When considering the potential anti-inflammatory mechanism of action of CBG, it should be mentioned that it is a PPARγ receptor agonist, and these have the ability to reduce inflammation (Atalay et al., 2019). Rosiglitazone and pioglitazone, which act as potent PPARγ agonists, are among a group of effective and used antidiabetic drugs (Han et al., 2017). Given that diabetes and hypertension are elements of the metabolic syndrome and are often comorbid conditions, it seems that CBG, due to its unique receptor mechanism of action, could even find application in the multidirectional therapy of the metabolic syndrome (Nachnani et al., 2021). Recent studies indicate that CBG therapy (30 mg/kg for 14 days) affects sphingolipid metabolism in the liver and plasma of rats subjected to high-fat and high-saccharose diet, which may promote liver protection against the development of insulin resistance (Bzdęga et al., 2023) (Table 3). In addition, CBG treatment at the same dose and duration showed beneficial effects on intramuscular phospholipid composition, altering the content of specific phospholipid subclasses and the fatty acid profile. These changes reduced the inflammatory response in the skeletal muscles of insulin-resistant rats fed a diet rich in fat and sucrose (Bielawiec et al., 2024) (Table 3). Similarly, Sztolsztener et al. (2024) showed that administration of CBG (30 mg/kg for 14 days) inhibited inflammation in the colon in rats which may be a potential protection against cancer development (Table 3).

### 4.5 Influence of cannabigerol on parameters in blood and body weight

There is a strong, complex and still not fully understood relationship between hypertension and metabolic syndrome. In the course of hypertension and in patients with cardiovascular risk, it is recommended to monitor and maintain appropriate parameters of lipid and carbohydrate metabolism (Mancia et al., 2023). To our knowledge, there are few data describing the effects of CBG on basic parameters of blood count, hemostasis, lipid profile or carbohydrate metabolism.

Studies have shown that ECS exerts control over many processes in the body, such as appetite regulation, energy balance and metabolism (Jager and Witkamp, 2014; van Eenige et al., 2018; Kurtov et al., 2024). Interactions between phytocannabinoids and the ECS can affect the metabolism of endogenous ligands (Bielawiec et al., 2020), so there is a reasonable suspicion that cannabinoids including CBG can affect the basal lipid or carbohydrate profile. Recent studies have shown that CBG at a dose of 1.33 mg/kg/day administered to rats for 90 days reduced body weight and triglycerides level (Polanska et al., 2023), while high doses of CBG 120–240 mg/kg stimulated food intake in rats (Brierley et al., 2016) (Table 3). In addition, CBG at doses of 0.66 and 1.33 mg/kg/day reduced platelet counts (Polanska et al., 2023), and in another study CBG inhibited platelet aggregation in rabbits, as well as in humans (*K*_*i*_ = 2.7 × 10^−4^ M), induced by adenosine diphosphate which may be important in preventing dangerous cardiovascular incidents associated with elevated BP (Formukong et al., 1989). Moreover, it is also worth mentioning that CBG was effective in reducing inflammation and thus protecting blood brain barrier cells subjected to oxygen-glucose deprivation, suggesting, the usefulness of CBG in ischemic stroke therapy (Stone et al., 2021).

### 4.6 Influence of cannabigerol on organs - Potential limitations

It is known that end-organ function/architecture is altered and deteriorated in the course of hypertension (Oparil et al., 2018). In opposition to the known beneficial anti-inflammatory, antioxidant properties of CBG, there are reports that prolonged exposure to CBG can cause changes in the liver. Hepatocytes after chronic (90 days), oral administration of CBG at doses of 0.66 and 1.33 mg/kg showed regressive changes - cytoplasmic granular changes with dispersed apoptotic cells, no changes were observed after CBG in the gastrointestinal tract (Polanska et al., 2023). Aljobaily et al. (2022) observed that low doses of CBG (2.46 mg/kg) are able to alleviate the symptoms of non-alcoholic fatty liver in mice while high doses (24.6 mg/day) can worsen liver damage (Table 3). Conversely, other authors have postulated that CBG (30 mg/kg for 14 days), by enhancing ceramide transport into the plasma, may prevent the development of hepatic steatosis in rats on high-fat and high-saccharose diet (Bzdęga et al., 2023). Interestingly, a recent study by Gao et al. (2024) found that cannabinol (CBN), cannabichromene (CBC) and CBD, but not CBG, can impair important liver detoxification mechanisms by inhibiting the pregnane X receptor (PXR) and constitutive androstane receptor (CAR) pathways. According to other reports, CBG at a dose of 15 mg/kg reduced plasma aspartate aminotransferase (AST) levels, but did not reduce hepatic steatosis in mice on a high-fat diet (Kogan et al., 2021). Sztolsztener et al. (2023) showed that CBG at low concentrations (5 µM) in hepatocytes exposed to palmitate and fructose reduces the concentration of transforming growth factor beta 1 (TGF-β1), which can accelerate regression of liver fibrosis and improve liver regeneration while high concentrations (30 µM) showed the opposite effect. Currently, to our knowledge, there are reports of various, often opposing dose-dependent effects of CBG on the liver, however, there is a lack of any data on the effects of CBG on the kidneys, blood vessels and heart.

### 4.7 Clinical studies

There are currently 10 studies registered on the ClinicalTrials.gov website for the phrase “cannabigerol”, 6 of which involve the administration of pure CBG (i.e., without any additives *etc.*). The purpose of the NCT05257044 study was to evaluate the effects of CBG (20 mg of CBG tincture) on stress, anxiety and cognitive function in general, while assessing possible side effects. Recently, the first results of the aforementioned study appeared Cuttler et al. (2024) showed that CBG reduced feelings of stress and anxiety, as well as had a beneficial effect on memory. The NCT05088018 study will determine the effects of CBG (orally 25 mg daily for 2 weeks, followed by 50 mg daily orally also for 2 weeks) on sleep and quality of life in war veterans. The NCT06115603 study will assess the usefulness of CBG (orally 80 mg daily for 2 weeks) in alleviating symptoms in patients with attention-deficit hyperactivity disorder (ADHD). The NCT05743985 study will evaluate the effects of taking CBG (orally 50 mg daily for 8 weeks) on the mental, physical and emotional wellbeing of healthy subjects, as well as on inflammation, while assessing side effects. The NCT06513507 study will evaluate the effects of CBG (50 mg daily for 8 weeks) on patients’ quality of life and rheumatoid arthritis symptoms, as well as inflammatory parameters. And participants in the study with the identifier NCT06638996 will undergo a series of examinations and tests to evaluate the effects of a single dose of CBG (50 mg) on stress anxiety, memory, salivary cortisol, electrodermal activity, HR, BP, pain tolerance and potential side effects.

The aforementioned studies mainly focus on CBG’s effects on nervous system function and cognitive function. The use of CBG in clinical trials for the aforementioned purposes, the growing interest in CBG-containing dietary supplements, coupled with studies showing that CBG can modify BP demonstrate the urgent need to comprehensively study the effects of CBG on the cardiovascular system and determine its safety and therapeutic potential.

## 5 Conclusion

**FIGURE 4:**
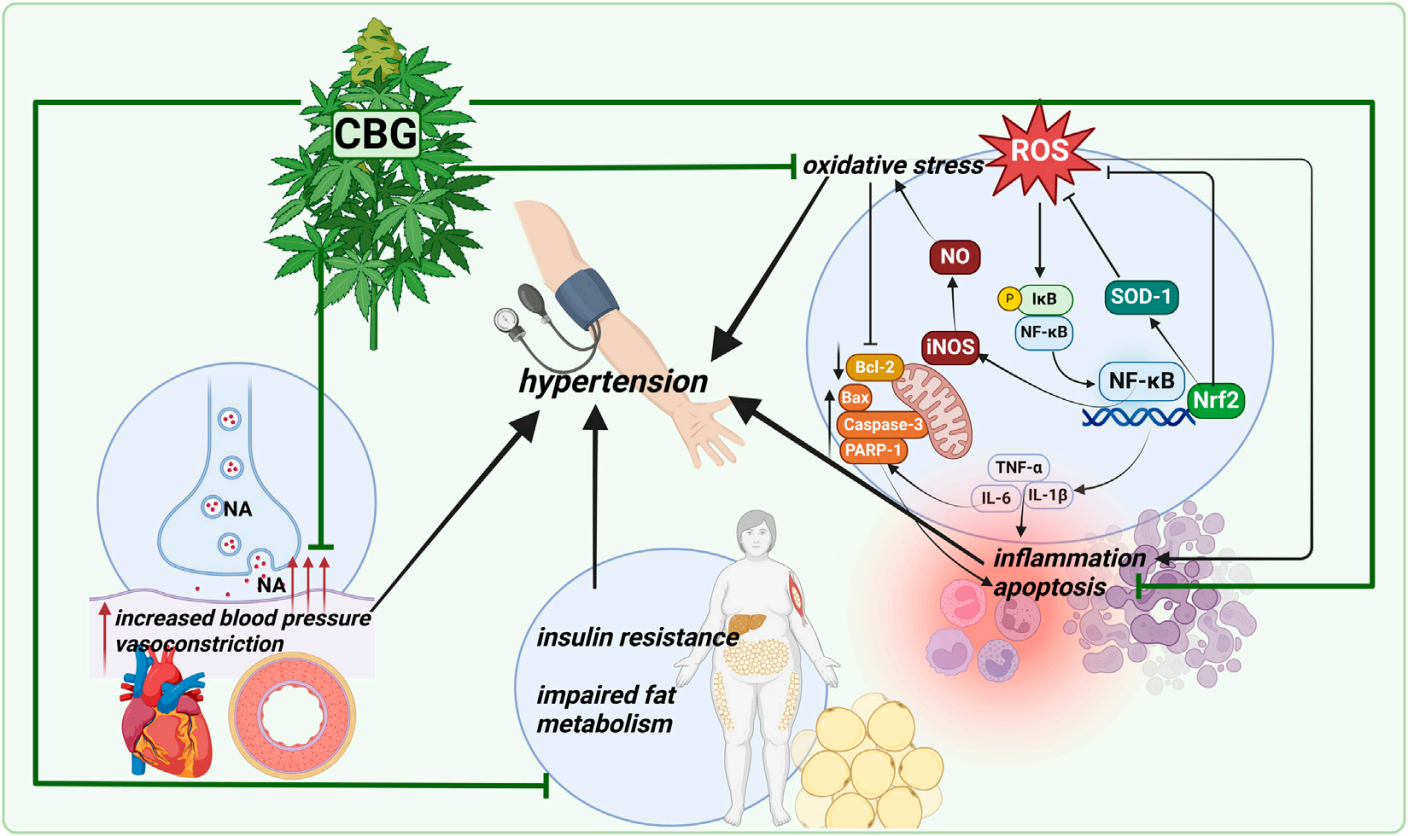
A summary of the likely effects of cannabigerol, which could be useful in the combating against hypertension. Abbreviations: Bax, Bcl-2-associated X protein; Bcl-2, B-cell lymphoma 2; CBG, cannabigerol; IκB, inhibitor of nuclear factor kappa B; IL-1β, interleukin 1 beta; IL-6, interleukin 6; iNOS, nitric oxide synthase; NA, noradrenaline; NF-κB, nuclear factor kappa-light-chain-enhancer of activated B cells; NO, nitric oxide; Nrf-2, nuclear factor erythroid 2-related factor 2; PARP-1, poly (ADP-ribose) polymerase-1; ROS, reactive oxygen species; SOD-1, superoxide dismutase 1; TNF-α, tumour necrosis factor alpha. Created with BioRender.

In conclusion, the effects of CBG described above, including BP lowering, anti-inflammatory and antioxidant effects, suggest that CBG may have a role in the treatment of diseases with elevated BP, including hypertension. However, there is still a lack of studies on the chronic administration of CBG and its effects on cardiovascular parameters in hypertension condition, which may be necessary to determine its safety and future studies on other indications. In addition, CBG, due to its specific receptor potential and reports of its potential action to improve tissue sensitivity to insulin, may find application in the treatment of metabolic syndrome. On the other hand, given reports of adverse effects of high doses of CBG on liver architecture and function, further studies are required to establish the safety profile of CBG. Figure 4 summarizes the likely effects of CBG, which could be useful in combating hypertension.

## Glossary

α_2_AR: alpha 2 adrenoceptor
2-AG: 2-arachidonoylglycerol
4-HHE: 4-hydroxyhexenal
4-HNE: 4-hydroxynonenal
5-HT_1A_: serotonin receptor type 1A
ADHD: attention-deficit hyperactivity disorder
AEA: N-arachidonoylethanolamine
AST: aspartate aminotransferase
BP: blood pressure
CAR: constitutive androstane receptor
CB-Rs: cannabinoid receptors
CB_2_-R: cannabinoid type 2 receptor
CBC: cannabichromene
CBD: cannabidiol
CBG: cannabigerol
CBN: cannabinol
COX-1/2: cyclooxygenase 1 and 2
ECS: endocannabinoid system
GPCRs: Gi-coupled G-protein coupled receptors
H_2_O_2_: hydrogen peroxide
HR: heart rate
IFN-γ: interferon gamma
IL: interleukin
*i.p.*: intraperitoneal
INOS: inducible nitric oxide synthase
*i.v.*: intravenous
LPS: lipopolysaccharide
MBP: mean blood pressure
MCP-1: monocyte chemoattractant protein 1
MDA: malondialdehyde
MethAEA: methanandamide
NA: noradrenaline
NF-κB: nuclear factor kappa-light-chain-enhancer of activated B cells
NO: nitric oxide
PARP-1: nitrotyrosine and Poly (ADP-ribose) polymerase
PEA: palmitoylethanolamide
PH: pulmonary hypertension
PPARγ: peroxisome proliferator-activated receptor gamma
PXR: pregnane X receptor
ROS: reactive oxygen species
*s.c.*: subcutaneous
SBP: systolic blood pressure
SHR: spontaneously hypertensive rat
SOD-1: superoxide dismutase-1
Δ^9^-THC: Δ^9^-tetrahydrocannabinol
TGF-β: transforming growth factor beta
TNF-α: tumor necrosis factor alpha
WKY: Wistar Kyoto
